# Use of Flavored E-Cigarettes and the Type of E-Cigarette Devices Used among Adults and Youth in the US—Results from Wave 3 of the Population Assessment of Tobacco and Health Study (2015–2016)

**DOI:** 10.3390/ijerph16162991

**Published:** 2019-08-20

**Authors:** Liane M. Schneller, Maansi Bansal-Travers, Maciej L. Goniewicz, Scott McIntosh, Deborah Ossip, Richard J. O’Connor

**Affiliations:** 1 Department of Health Behavior, Roswell Park Comprehensive Cancer Center, Buffalo, NY 14263, USA; 2 Department of Public Health Sciences, University of Rochester Medical Center, Rochester, NY 14642, USA

**Keywords:** electronic cigarettes, vaping, tobacco, flavors, regulation, population

## Abstract

The United States (U.S.) Food and Drug Administration has expressed concern about flavored e-cigarettes (e.g., JUUL brand) because they are appealing to youth who may be unaware that the product is addictive. The Population Assessment of Tobacco and Health Study Wave 3 provided data on flavor categories, type of e-cigarette product, and smoking status among past 30-day youth and adult e-cigarette users in the US. Most past 30-day youth and adult users reported using only one flavor category, with fruit (53% youth, 31% adult) being the most commonly reported category. Adults were far more likely to report using tobacco flavor alone, compared to any other individual flavor category or flavor category combinations (OR: 21.08, 95%CI: 5.92, 75.12). Whereas, youth were more likely to report using multiple flavor categories (OR: 2.03, 95%CI: 1.55, 2.65), with the most reported pairing being fruit and candy (36%). The variety of flavors on the market appeals to consumers of all ages. Although most past 30-day e-cigarette users reported only one flavor category, non-tobacco flavors were far more common among youth. Differences in flavor preferences among adult versus youth vapers may have implications for the role of flavors in both the initiation of youth vaping and adult vaping for smoking cessation.

## 1. Introduction

The United States (U.S.) Food and Drug Administration (FDA) was given the authority to regulate all tobacco products, including electronic cigarettes (e-cigarettes), following the passing of the Deeming rule in August 2016 [1]. Per these regulations, the FDA now has the authority to regulate the presence of ‘characterizing flavors’ in e-cigarettes, as was done with cigarettes through the Family Smoking Prevention and Control Act of 2009 [1]. The wide variety of flavored e-cigarettes is enticing to both new consumers and established cigarette smokers [2,3,4,5,6]. In 2018, 4.9% of U.S. middle schoolers and 20.8% of U.S. high schoolers used an e-cigarette in the past 30-days [7], while 2.8% of U.S. adults were current e-cigarette users in 2017 [8]. Former FDA Commissioner Gottlieb characterized e-cigarette use among the youth population as an epidemic [9]. Consumers must be 18 years or older to purchase e-cigarette products in most states, but underage sales are occurring in retailers and online [9]. As a result, the FDA is investigating policies that would allow e-cigarettes to be available and attractive for adults trying to transition from cigarette smoking, but less accessible and appealing to youth and young adult nonsmokers [9].

The sensory effect of e-cigarette use (vaping) is created by a combination of taste, smell, and airway stimulation [10,11]. Many e-cigarette characteristics, including type of device, electrical power source, nicotine content, and e-liquid flavors, influence the sensory effects of e-cigarettes [11,12,13,14,15,16,17]. The addition of flavorings is shown to reduce the harshness of the nicotine taste in many tobacco products, which allows for increased inhalation, increased nicotine delivery, and increased appeal [18,19,20,21,22,23]. Established cigarette smokers looking to quit may use e-cigarettes as a cessation tool in part because of appealing flavors [5]. However, the FDA has expressed concern that certain flavored e-cigarettes (e.g., JUUL brand) are appealing to youth who may be unaware of the products’ addictiveness and otherwise may have never tried a nicotine product [24]. According to data from 2013 to 2014, 81% of current youth e-cigarette users in the U.S. reported that a main reason for use was the availability of flavors [2]. Sweeter flavors like fruit and candy are preferred among young adults 18–30 years of age, while flavors like tobacco or clove/spice are not as appealing [11,25,26,27].

Previous studies have looked at why consumers use flavored e-cigarettes [2,5], the health perceptions of flavored e-cigarettes [28], the effect of flavored e-cigarettes on youth and young adult tobacco use [3], flavored e-cigarette use among youth versus adults [29,30,31], and common flavors and number of flavors used by consumers [32]. However, newer data from the Population Assessment of Tobacco and Health (PATH) Study allows researchers to assess the use of different flavors, such as tobacco flavor, in combination with other flavors, as questionnaires were updated to keep up with the changing e-cigarette marketplace. Policy change would likely only affect the youth (12–17 years) population, and a better understanding of the flavor preferences among the youth and adult populations could be informative in the banning of characterizing flavors. Although a certain flavor(s) (e.g., tobacco versus a sweeter flavor, or the ability to ‘mix and match’ flavors) could be attractive to an adult smoker trying to quit, it may also be enticing to a teen who has never used an e-cigarette or even a nicotine product. This analysis aimed to assess the use of flavored e-cigarettes and the association of the number of flavors reported with the type of e-cigarette product used, vaping frequency, and smoking status, among nationally-representative samples of adults and youth in the U.S. from Wave 3 (W3) of the PATH Study.

## 2. Materials and Methods

### 2.1. Study Design and Population

The PATH Study is a nationally representative, longitudinal cohort study of tobacco use and its health effects developed by the National Institutes of Health (NIH) and the FDA [33]. Its sample population aims to represent all of the noninstitutionalized U.S. population, 12 years of age or older. The PATH Study W3 Youth (12–17 years old) and Adult (18+ years old) public-use data files were analyzed. Data were collected between October 19, 2015 and October 23, 2016. W3 included measures that allowed participants to identify use of specific flavor categories for each product used, including e-cigarettes. There were 28,148 total adult cases and 11,814 total youth cases. Information about the PATH Study design and methods can be found elsewhere [34].

This study reports W3 cross-sectional estimates from 415 youth (12–17 years) participants and 2123 adult (18 years and older) participants who reported use of an e-cigarette, not exclusive to the use of other electronic nicotine delivery systems (ENDS) or other tobacco products, in the past 30-days. Analyses were done to identify the most prevalent e-liquid flavor categories used individually or in combination with other flavor categories, and the association of the number of flavor categories reported with the type of e-cigarette product used, vaping frequency and smoking status taking into account participant demographics (see Table 1).

### 2.2. Nicotine Use Behavior

Participants were asked “In the past 30 days, were/was any of the e-cigarettes/e-cigarette cartridges/e-liquid you used flavored to taste like tobacco, menthol or mint, clove or spice, fruit, chocolate, an alcoholic drink, a nonalcoholic drink, candy, dessert or other sweets, or some other flavor? Choose all that apply.” The response options provided flavor categories including tobacco, menthol/mint, clove/spice, fruit, chocolate, an alcoholic drink, a non-alcoholic drink, candy/desserts/other sweets, or some other flavor. Tobacco flavor was of interest because any ban on characterizing flavors probably would not include tobacco flavor. In addition, the number of flavors was of interest because the wide range of flavors that can be ‘mixed and matched’ are appealing to consumers of all ages.

Participants were also categorized based on the type of e-cigarette product they reported using (e.g., open/refillable versus closed/not refillable). Participants were asked, “Can you refill your e-cigarette with ‘e-liquid’?” Furthermore, participants were categorized on vaping frequency (e.g., daily versus non-daily). They were asked, “On how many of the past 30 days did you use an e-cigarette?” If they reported 30 days, then they were considered daily vapers, and if they reported less than 30, then they were considered non-daily vapers.

Cigarette smoking status (e.g., Never, Former, Someday, and Every day) was assessed. Derived variables for smoking status were provided for the adult population. Among the youth population, only an “Ever Smoker” variable, as well as a “Current smoker” variable, was provided. Therefore, if the participant was not marked as an ever smoker, then they were considered a never smoker. If participants were marked as an ever smoker but not marked as a current smoker, then they were considered a former smoker. Someday and every day smoking status was determined if they were marked as a current smoker, and by the following question, “In the past 30 days, on how many days did you smoke cigarettes?” Participants who reported 30 days were considered every day smokers, and participants who reported less than 30 days were considered someday smokers. Finally, participants who currently smoked were asked “In the past 12 months have you tried to quit smoking completely?” as well as “Do you plan to quit smoking for good?”

### 2.3. Statistical Analysis

Prevalence of top flavor combinations, as well as flavors used alone, was assessed among youth and adult past 30-day e-cigarettes users. Analyses were conducted using W3 replicate weights and balanced repeated replication methods with Fay’s adjustment of 0.3 in Stata 14 software (2011, StataCorp LLC, College Station, TX, USA). Descriptive statistics and logistic regressions were used to look at the association of the type of ENDS flavor category used and the number of ENDS flavor categories used among past 30-day youth and adult e-cigarette users with demographic and nicotine use behaviors. In addition, the association of past combustible cigarette quit attempts and intentions with the type of ENDS flavor category used and the number of ENDS flavor categories used among past 30-day adult e-cigarette users was assessed. Weighted percentages, adjusted odds ratios (AOR) and 95% confidence intervals (95%CI) are reported. Models were adjusted for gender, race/ethnicity, and type of e-cigarette device used (open versus closed). Models assessing the association of quit attempts and quit intentions were adjusted for covariates listed above, as well as time to first nicotine product of the day.

## 3. Results

### 3.1. Demographic Characteristics of Past 30-Day Adult and Youth E-Cigarette Users

The adult and youth vaping populations did not differ in gender or race/ethnicity. However, significant differences between the adult and youth populations were seen in the number of flavor categories reported (*p* < 0.0001), type of e-cigarette device (*p* < 0.0001), vaping frequency (*p* = 0.0002), and smoking status (*p* < 0.0001; see Table 1). Among youth who reported using an e-cigarette on at least one day in the past 30 days, 53.9% were male, 78.5% were Non-Hispanic White, 85.9% used an open, refillable e-cigarette, 93.2% were non-daily vapers, and 38.3% were never cigarette smokers (see Table 1). Among adult past 30-day e-cigarette users, 55.2% were male, 79.7% were non-Hispanic White, 76.7% reported using an open, refillable e-cigarette device, 97.1% were non-daily vapers, and 30.7% were former cigarette smokers (see Table 1).

### 3.2. Flavor Category Preferences of Past 30-Day Adult and Youth E-Cigarette Users

Among the 226 youth participants (51.6%) who reported using only one flavor category, the top three flavor categories reported included fruit (52.8%), candy/desserts/other sweets (24.4%), and menthol/mint (10.8%; see Table 2). Nearly half (45.5%) of youth participants reported two or more flavor categories. Fruit flavor was in nine out of the top 10 flavor combinations while candy/desserts/other sweets appeared in seven out of the top 10 flavor combinations. Clove/spice was the least popular individually reported flavor category (0.8%) and did not appear in any of the top 10 flavor combinations (see Table 2).

There were 1809 adult participants (66.6%) who reported using only one flavor category. The top three flavor categories included fruit (30.8%), tobacco (24.5%), and candy/desserts/other sweets (18.2%; see Table 2). There were 944 adult participants (32.1%) who reported two or more flavor categories. Similar to the youth population, fruit flavor was the most common flavor category to be reported alone and with another flavor(s), while clove/spice was the least popular individually reported flavor category and did not appear in any of the top 10 flavor combinations. In addition, the alcoholic drink flavor category did not appear in the top 10 flavor combinations (see Table 2).

### 3.3. Association of Demographic and Nicotine Use Behavior Characteristics with Type of Flavor Category among Past 30-Day Adult and Youth E-Cigarette Users

Adjusting for gender, race/ethnicity, dual use of cigarettes, and device type, youth had 21 times greater odds (96%CI: 5.92, 75.12) of using a non-tobacco flavor in their e-cigarette. Furthermore, after the adjustment for the demographic and nicotine use behavior characteristics, consumers who identified as an Other Race/Ethnicity were more likely to use a non-tobacco flavor vaping product (AOR: 2.08, 95%CI: 1.33, 3.24). When examining the types of devices, consumers who use an open device that can be refilled were more likely to report a non-tobacco flavor category or some combination of flavor categories versus tobacco alone, compared to a closed, non-refillable device (AOR: 5.42, 95%CI: 4.07, 7.22), after adjustment for age, gender, and race/ethnicity. Consumers who were past 30-day vapers and a current smoker were less likely to report a non-tobacco flavor category or some flavor combination versus a tobacco flavor alone in comparison to exclusive vapers (AOR: 0.67, 95%CI: 0.48, 0.93), after adjustment (see Table 3). In addition, after the adjustment for age group, gender, race/ethnicity, and ENDS device, current vapers who were never smokers were more likely to report a non-tobacco flavor category or some flavor combination versus a tobacco flavor alone in comparison to current smokers (AOR: 40.1, 95%CI: 2.76, 581.5). However, there were few never smokers who reported using tobacco flavor alone, making this point estimate unstable.

### 3.4. Association of Demographic and Nicotine Use Behavior Characteristics with the Number of Flavor Categories Among Past 30-Day Adult and Youth E-Cigarette Users

**Figure 1:**
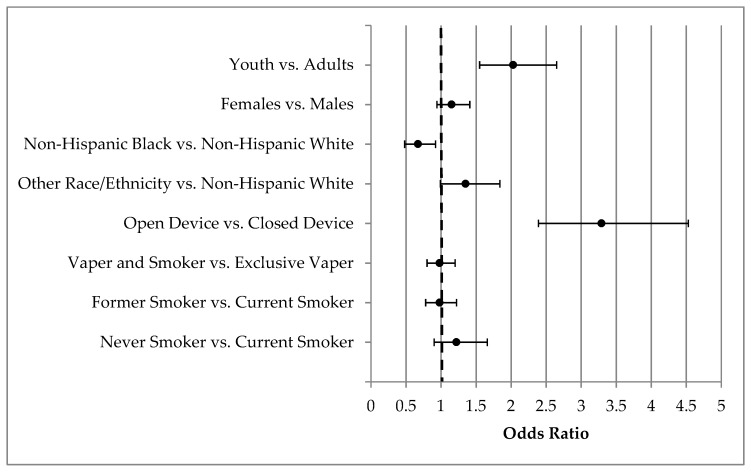
Association of demographic and nicotine use behavior characteristics with use of two or more ENDS flavor categories vs. one flavor category.

The youth population was more likely to report using two or more flavors compared to the adult population (AOR: 2.03, 95%CI: 1.55, 2.65). In contrast, past 30-day vapers who were Non-Hispanic Black were less likely to report two or more flavors compared to those identifying as Non-Hispanic White (AOR: 0.67 95%CI: 0.48, 0.92). Finally, those who used an open, refillable device were more likely to report two or more flavors compared to those using a closed, non-refillable device (AOR: 3.29, 95%CI: 2.39, 4.53; see Figure 1).

### 3.5. Association of Demographic Characteristics and Ever Smoking Status with the Type of Flavor Category Among Past 30-Day Adult E-Cigarette Users

The association of demographic characteristics and ever smoking status with type of flavor category was assessed for the adult population only, due to sample size for the youth population. Adult past 30-day adult e-cigarette users who were older than 18 to 24 years of age were less likely to report using a non-tobacco flavor category or some flavor combination versus tobacco alone (25–34 years: AOR: 0.37, 95%CI: 0.19, 0.71; 35–54 years: AOR: 0.13, 95%CI: 0.08, 0.23; 55 years and older: AOR: 0.08, 95%CI: 0.05, 0.14), after adjustment, relative to the 18–24 group. In contrast, after adjustment, females (AOR: 1.75, 95%CI: 1.29, 2.36) and Non-Hispanic Blacks (AOR: 2.65, 95%CI: 1.37, 5.12) were more likely to report using a non-tobacco flavor category or some flavor combination versus tobacco alone. Similar to the above analysis, consumers who reported using an open, refillable ENDS device were more likely to report using a non-tobacco flavor category or some flavor combination versus tobacco alone (AOR: 5.31, 95%CI: 3.84, 7.34), after adjustment. Finally, among the past 30-day adult e-cigarette users who were ever smokers, those who were former smokers were about one and a half times more likely (95%CI: 1.00, 2.12) to report using a non-tobacco flavor category or some flavor combination versus tobacco alone compared to current smokers, after adjustment, though this association was of borderline significance (see Table 4).

### 3.6. Association of Type of Flavor Category and Number of Flavor Categories with Past Cigarette Smoking Quit Attempts and Cigarette Smoking Quit Intentions Among Adult Past 30-Day Vapers and Current Smokers

For adults who were past 30-day users of e-cigarettes and current smokers (dual users), there was no association between type of ENDS flavor category or flavor combination versus tobacco flavor alone and a cigarette quit attempt in the past 12 months (AOR: 1.41, 95%CI: 0.97, 2.05). Conversely, dual users who reported using two or more flavors were more likely to have made a cigarette quit attempt in the past 12 months compared to those who reported using only one ENDS flavor (AOR: 1.49, 95%CI: 1.09, 2.06). Finally, there was no association between type of flavor category (AOR: 1.36, 95%CI: 0.83, 2.22) or number of flavor categories reported (AOR: 1.07, 95%CI: 0.72, 1.60) and cigarette smoking quit intentions among adult past 30-day dual users.

## 4. Discussion

Findings from the PATH Study W3, a nationally representative, longitudinal cohort study in the U.S., indicate that past 30-day youth vapers are substantially more likely than adults to be using a non-tobacco flavor vape. In addition, youth are significantly more likely to use more than one flavor, and to use combinations that do not involve tobacco. Fruit flavor was commonly used, alone and in combination with other flavor categories, among both youth and adult populations. However, the preference for tobacco flavor was more common among the adult population. Finally, an open, refillable system appears to be associated with the use of a greater variety of flavors. Previously, Schneller, et al. [32] identified that fruit flavor was the most common flavor category reported alone and in combination among past 30-day youth vapers followed by candy/other sweets using the PATH Study Wave 2 (W2; 2014–2015). The analyses presented in this paper using the next wave of PATH data (2015–2016) showed similar results, but with slightly fewer youth reporting fruit alone (55% in W2 and 53% in W3) and slightly more reporting candy/other sweets alone (21% in W2 and 24% in W3). However, differences were seen in the individual flavor preference among adults: at W2, menthol/mint was the most common flavor reported alone followed by fruit, while at W3, most adults reported fruit followed by tobacco flavor, and menthol/mint was the fourth most common flavor reported alone. This difference may partially be due to the change in the PATH Study questionnaire. At W2, tobacco flavor was not listed as a choice, but was added at W3 along with non-alcoholic beverage. Many similar flavor combinations appeared among the youth and adult populations with slight differences in prevalence, but this could also be a result of the additional flavor options at W3.

A similar study was conducted by Sonjei et al. [35] using PATH W2 data. Sonjei et al. [35] also identified that fruit and candy flavored e-cigarettes were most common among youth and young adults, while tobacco flavored e-cigarettes were most common among older adult e-cigarette users. In addition, never smokers were less likely to use a tobacco flavored e-cigarette. Finally, Sonjei et al. [35] found that youth and young adults were more likely to report using two or more flavors. However, one difference to note between the study by Sonjei et al. [35], the study by Schneller, et al. [32], and this current study is that Sonjei et al. [35] assessed three age groups, adolescents, young adults, and older adults, while the other two studies only assessed two, youth and adults.

The current study found that past 30-day vapers who were former smokers were more likely to report non-tobacco flavors compared to current smokers. In addition, vapers who reported two or more flavor categories were more likely to have made a quit attempt in the past 12 months, while flavor (tobacco alone versus non-tobacco or flavor combination) and number of flavor categories reported did not seem to be associated with quit intent. E-cigarettes may be used by established cigarette smokers as a cessation tool in part because of appealing flavors [5]. Former smokers who have completely switched to an e-cigarette are more likely to have transitioned from a tobacco flavored product to a non-tobacco flavored product overtime [36]. Therefore, the banning of characterizing flavors may prevent youth initiation, but this may also deter smokers from quitting with the help of a flavored e-cigarette product [37].

Although the PATH study has strong external validity, internal validity, and retention rates, there are some limitations to note. First, although PATH has a large overall sample size, our statistical power to assess associations among past 30-day youth and adult e-cigarette users was limited. Second, the flavor category data are reported based on a “check all that apply” list. Therefore, we cannot distinguish if multiple flavor categories are used together or individually. Third, the flavor list provided to participants is of flavor categories, which does not allow us to look at the use of multiple individual flavors that might fall under the same category. Fourth, we cannot identify the chemical additives used to create the flavors listed, as various chemical additives could be used to create a single flavor profile and may differ between brands. Finally, we did not assess the presence of nicotine in the e-cigarettes, which should be done in the future. Nicotine levels could provide public health implications as some adult smokers use e-cigarettes as a cessation tool [5], while many youth are misinformed and unaware of the nicotine concentration of e-cigarettes [36].

## 5. Conclusions

These findings build upon previous research [11,25,26,27], and reinforce the FDA concerns about the role of flavors in youth vaping appeal, as many youth are unaware of the addictiveness of e-cigarettes [36]. The addition of flavorings to a variety of tobacco products have been shown to reduce the harshness of the nicotine taste and promotes continued use [18,19,20,21,22,23]. Previous research has shown that sweet flavors such as fruit or dessert are more satisfying and act as a positive reinforcer among the young adult population (18–30 years) [26]. With a multitude of flavors and flavor combinations available on the market, consumers of all ages have many options that enable them to tailor their choices to their individual preferences and thus may encourage initial and continued use. Although most past 30-day e-cigarette users reported using only one flavor category, non-tobacco flavors are far more common among youth than adults. Results from this study identified differences in flavor preferences among adult versus youth vapers that may imply roles for flavors in both the initiation of youth vaping and in adult vaping, including for smoking cessation.

## Figures and Tables

**Table 1:** Demographic characteristics of past 30-day adult and youth e-cigarette users who provided flavor data in the Population Assessment of Tobacco and Health (PATH) Study Wave 3, 2015–2016.

Characteristics Assessed at W3	Adult Past 30-day ENDS User	Youth Past 30-day ENDS User	*p*-Value
	*N* = 2784	*N* = 433	
Gender, *N* (%)			0.6465
Male	1484 (55.2)	230 (53.9)	
Female	1299 (44.9)	202 (46.1)	
Race/Ethnicity, *N* (%)			0.3568
Non-Hispanic White	2095 (79.7)	313 (78.5)	
Non-Hispanic Black	303 (10.5)	41 (9.5)	
Other groups	319 (9.8)	67 (12.0)	
Number of Flavor Categories, *N* (%)			<0.0001
1 Flavor Category	1809 (66.6)	226 (51.6)	
2+ Flavor Categories	944 (32.1)	194 (45.5)	
Don’t Know	31 (1.3)	13 (2.9)	
Flavor Category, *N* (%)			<0.0001
Tobacco alone	383 (16.5)	13 (2.7)	
All other flavors and flavor combinations	2370 (83.5)	407 (97.3)	
ENDs Device, *N* (%)			<0.0001
Open	2128 (76.7)	325 (85.9)	
Closed	632 (23.3)	54 (14.1)	
Dual Use of Cigarettes, *N* (%)			<0.0001
Exclusive Vaper	796 (37.6)	283 (66.3)	
Vaper and Smoker	1403 (62.4)	145 (33.7)	
Smoking Frequency, *N* (%)			<0.0001
Never	197 (6.9)	167 (38.3)	
Former	599 (30.7)	116 (28.0)	
Current	1403 (62.4)	145 (33.7)	

ENDS: electronic nicotine delivery systems.

**Table 2:** Prevalence of ENDS flavor categories and flavor combinations among adult and youth respondents that reported using only one in the PATH Study Wave 3, 2015–2016.

Most Common Flavors			
Adult Past 30-day ENDS User *N* = 1809		Youth Past 30-day ENDS User *N* = 226	
	**%** **(LB, UB)**		**%** **(LB, UB)**
Fruit	30.8 (28.1, 33.6)	Fruit	52.8 (45.4, 60.1)
Tobacco	24.5 (21.6, 27.6)	Candy/desserts/other sweets	24.4 (19.9, 29.7)
Candy/desserts/other sweets	18.2 (15.7, 21.1)	Menthol/Mint	10.8 (7.0, 16.2)
Menthol/Mint	17.9 (15.9, 20.1)	Tobacco	5.1 (3.0, 8.4)
Other flavor	3.1 (2.2, 4.3)	Chocolate	3.0 (1.1, 7.8)
Non-Alcoholic Beverage	2.5 (1.9, 3.5)	Alcoholic Beverage	1.1 (0.2, 5.3)
Alcoholic Beverage	1.5 (0.9, 2.5)	Non-alcoholic Beverage	1.0 (0.3, 3.2)
Chocolate	0.9 (0.6, 1.6)	Other flavor	1.0 (0.3, 3.2)
Clove/Spice	0.6 (0.3, 1.1)	Clove/Spice	0.8 (0.2, 3.1)
**Most Common Flavor Combinations**			
**Adult Past 30-day ENDS User *N* = 944**		**Youth Past 30-day ENDS User *N* = 194**	
	**%** **(LB, UB)**		**%** **(LB, UB)**
Candy/desserts/other sweets and some other flavor	33.4 (29.9, 37.1)	Fruit and Candy/desserts/other sweets	35.6 (29.1, 42.6)
Menthol/Mint and Fruit	7.6 (5.6, 10.1)	Fruit, Non-Alcoholic Beverage and Candy/desserts/other sweets	8.1 (4.8, 13.3)
Menthol/Mint, Fruit, and Candy/desserts/other sweets	6.7 (4.9, 9.1)	Fruit, Chocolate, and Candy/desserts/other sweets	5.6 (3.3, 9.3)
Tobacco and Menthol/Mint	4.0 (2.5, 6.1)	Menthol/Mint, Fruit and Candy/desserts/other sweets	3.6 (1.8, 7.1)
Fruit, Non-Alcoholic Beverage, and Candy/desserts/other sweets	3.7 (2.8, 5.0)	Fruit, Alcoholic Beverage, and Candy/desserts/other sweets	3.6 (1.5, 8.2)
Fruit, Chocolate, and Candy/desserts/other sweets	3.4 (2.2, 5.4)	Menthol/Mint and Fruit	3.6 (1.6, 7.8)
Tobacco and Fruit	3.2 (2.1, 4.9)	Menthol/Mint, Fruit, Non-Alcoholic Beverage, and Candy/desserts/other sweets	2.5 (0.9, 6.8)
Menthol/Mint and Candy/desserts/other sweets	2.6 (1.7, 4.2)	Tobacco, Fruit, and Candy/desserts/other sweets	2.3 (0.9, 5.6)
Tobacco, Fruit, and Candy/desserts/other sweets	2.4 (1.4, 4.1)	Fruit and Chocolate	2.0 (0.6, 6.5)
Tobacco and Candy/desserts/other sweets	1.9 (1.1, 3.2)	Tobacco and Menthol/Mint	2.0 (0.6, 6.4)

**Table 3:** Association between ENDS tobacco flavor use and cigarette use among past 30-day adult and youth e-cigarette users in the PATH Study Wave 3, 2015–2016.

	Tobacco Flavor Alone	All Other Flavors and Flavor Combinations		
	*N* = 396	*N* = 2777		
	*N* (%)	*N* (%)	Crude	Adjusted ^a^
Age Group				
Adult	383 (98.8)	2370 (91.9)	Ref	Ref
Youth	13 (1.2)	407 (8.2)	**7.14 (4.03, 12.7)**	**21.08 (5.92, 75.12)**
Gender				
Male	214 (58.6)	1479 (54.6)	Ref	Ref
Female	182 (41.4)	1296 (45.4)	1.17 (0.90, 1.54)	1.40 (1.07, 1.83)
Race/Ethnicity				
Non-Hispanic White	316 (82.5)	2059 (79.0)	Ref	Ref
Non-Hispanic Black	40 (10.9)	302 (10.5)	1.01 (0.59, 1.73)	1.40 (0.71, 2.76)
Other	36 (6.6)	342 (10.5)	**1.66 (1.09, 2.53)**	**2.08 (1.33, 3.24)**
ENDS Device				
Closed	202 (51.1)	471 (17.3)	Ref	Ref
Open	184 (48.9)	2247 (82.7)	**5.00 (3.84, 6.51)**	**5.42 (4.07, 7.22)**
Dual Use of Cigarettes				
Exclusive Vaper	92 (27.6)	968 (42.4)	Ref	Ref
Vaper and Smoker	257 (72.4)	1277 (57.6)	**0.52 (0.38, 0.70)**	**0.67 (0.48, 0.93)**

NOTE: Bolded text indicates statistical significance (*p* < 0.05); ^a^: Adjusted for age group, gender, race/ethnicity, and ENDS device.

**Table 4:** Association between ENDS tobacco flavor use and former smoking status among past 30-day adult e-cigarette users in the PATH Study Wave 3, 2015–2016.

	Tobacco Flavor Alone	All Other Flavors and Flavor Combinations		
	*N* = 383	*N* = 2187		
	*N* (%)	*N* (%)	Crude	Adjusted ^a^
Age				
18–24 years	45 (6.3)	1086 (32.9)	Ref	Ref
25–34 years	63 (18.7)	588 (28.5)	**0.29 (0.18, 0.47)**	**0.37 (0.19, 0.71)**
35–54 years	167 (44.2)	513 (27.0)	**0.12 (0.08, 0.17)**	**0.13 (0.08, 0.23)**
55 years and older	108 (30.8)	183 (11.53)	**0.07 (0.05, 0.11)**	**0.08 (0.05, 0.14)**
Gender				
Male	207 (58.5)	1261 (54.7)	Ref	Ref
Female	176 (41.5)	1108 (45.3)	1.17 (0.89, 1.54)	**1.75 (1.29, 2.36)**
Race/Ethnicity				
Non-Hispanic White	308 (82.8)	1764 (79.0)	Ref	Ref
Non-Hispanic Black	37 (10.6)	265 (10.6)	1.04 (0.59, 1.83)	**2.65 (1.37, 5.12)**
Other	35 (6.6)	278 (10.4)	1.64 (1.06, 2.51)	1.34 (0.77, 2.33)
ENDS Device				
Closed	199 (51.0)	423 (17.6)	Ref	Ref
Open	182 (49.0)	1931 (82.4)	**4.87 (3.70, 6.41)**	**5.31 (3.84, 7.34)**
Ever Smoker Status				
Current	252 (73.1)	1138 (65.5)	Ref	Ref
Former	83 (26.9)	513 (34.5)	**1.43 (1.04, 1.96)**	**1.46 (1.00, 2.12)**

NOTE: Bolded text indicates statistical significance (*p* < 0.05); ^a^: Adjusted for age group, gender, race/ethnicity, and ENDS device.
